# Platinum: a database of experimentally measured effects of mutations on structurally defined protein–ligand complexes

**DOI:** 10.1093/nar/gku966

**Published:** 2014-10-16

**Authors:** Douglas E.V. Pires, Tom L. Blundell, David B. Ascher

**Affiliations:** Department of Biochemistry, University of Cambridge, Cambridge CB2 1GA, UK

## Abstract

Drug resistance is a major challenge for the treatment of many diseases and a significant concern throughout the drug development process. The ability to understand and predict the effects of mutations on protein–ligand affinities and their roles in the emergence of resistance would significantly aid treatment and drug design strategies. In order to study and understand the impacts of missense mutations on the interaction of ligands with the proteome, we have developed Platinum (http://structure.bioc.cam.ac.uk/platinum). This manually curated, literature-derived database, comprising over 1000 mutations, associates for the first time experimental information on changes in affinity with three-dimensional structures of protein–ligand complexes. To minimize differences arising from experimental techniques and to directly compare binding affinities, Platinum considers only changes measured by the same group and with the same amino-acid sequence used for structure determination, providing a direct link between protein structure, how a ligand binds and how mutations alter the affinity of the ligand of the protein. We believe Platinum will be an invaluable resource for understanding the effects of mutations that give rise to drug resistance, a major problem emerging in pandemics including those caused by the influenza virus, in infectious diseases such as tuberculosis, in cancer and in many other life-threatening illnesses.

## INTRODUCTION

Mutations can result in a range of changes to protein function by altering its stability and affinity for binding partners including other proteins and peptides, nucleic acids and small molecules. The strong selective pressure imposed by small molecule drugs on many quickly evolving systems, including viruses, bacteria and human cancer, can lead to the rapid development of resistance (1–4). While rapid and cheaper DNA sequencing has allowed these mutations to be quickly identified in members of large populations (5,6), the significance and characterization of any novel polymorphisms currently requires time-consuming and costly experiments.

Ligand-binding affinity data for proteins have been an essential source of information for understanding the effects of polymorphisms in disease (7), in addition to identifying those that result in the development of resistance, an increasingly significant problem (8,9). Efforts to link missense mutations to the development of resistance in specific diseases, for example in cancer, tuberculosis and HIV (10–12), have highlighted the importance of these mutations. However, the understanding of the effects of mutations on ligand binding will help expand our knowledge of mechanisms of action, allowing extrapolations to novel mutations and systems. Awareness of these changes is also an essential step toward more effective, personalized and targeted treatment strategies. For example, the resistance profile of emerging influenza strains has been of significant interest to ensure that appropriate antiviral therapeutics can be rapidly administered in the event of an outbreak (13).

Databases that have linked non-synonymous single nucleotide polymorphisms (nsSNPs) with structural information and experimentally measured changes in thermodynamic data (14–16) have enabled the development of computational approaches to evaluate missense mutations (17–19) for their effects on protein stability and binding to protein and nucleic acid partners, expanding our understanding of their roles in disease. Despite its relevance and potential to support studies on emerging phenomena such as drug resistance, no such information is readily available to interrogate the effects of mutations on ligand-binding affinity.

A compilation of small molecule structural and affinity data for wild-type and mutant proteins would therefore be a valuable resource for developing methods to elucidate mechanisms behind ligand binding and to predict the effects of mutations. In order to fill this gap, we have designed and implemented a freely accessible database, called Platinum, which compiles and associates small molecule affinity data with structural information, experimental methods and conditions, and ligand properties. Furthermore, we have provided a web interface to facilitate searching the database, sorting, visualizing and downloading the results (http://structure.bioc.cam.ac.uk/platinum).

## MATERIALS AND METHODS

### Data acquisition and curation

The scientific literature available in PubMed was mined in order to select papers containing structural information of protein–ligand complexes and affinity data for complexes of both wild-type and mutant proteins.

An initial pool of papers was compiled for manual inspection by selecting those with mutation information associated with deposited protein–ligand complexes in the RCSB Protein Data Bank (20). In order to do so, paper titles and abstracts were filtered using regular expression matching in order to identify candidates for manual curation. The set of terms used on the regular expressions included root words of ‘mutation’, ‘resistance’ and mutation codes in the format <*WT-Res*><*Res-Num*><*MT-Res*>, where *WT-Res* is the one-letter code for the wild-type, *Res-Num* is the residue number and *MT-Res* is the one-letter code for the mutant residue.

This procedure identified over 1000 papers, which were manually evaluated against filtering criteria that include requiring affinity data for the construct for which the three-dimensional structure had been determined.

To minimize differences arising from experimental techniques and to be able to compare directly binding affinities (21), we considered only affinity changes measured by the same group, using the same technique and conditions, and with the same amino-acid sequence used to determine the three-dimensional structure, providing a direct link between protein structure, how a ligand binds and how mutations alter the affinity of the ligand for the protein. EC50/IC50 measurements in the absence of the Michaelis constant were not considered due to their inherent dependency on experimental conditions, and incompatibility with binding affinities. Only data from techniques that directly measure the ligand affinity to a protein were entered into Platinum, since indirect methods such as cellular assays have many confounding factors and were therefore discarded.

Approximately 20% of the identified papers matched our criteria and were manually curated to obtain data that would reflect the effects of mutations on protein–ligand affinity. Over 1000 different affinity data points were manually collated from 182 papers indexed by PubMed. Additional details regarding the experimental techniques and conditions were also entered into Platinum. The PDB-coordinate files for 250 different protein–ligand complexes were downloaded from the RCSB Protein Data Bank, pre-processed and used to supply additional information regarding the protein.

In order to account for additional consequences of the mutations, the predicted effects of single point mutations on protein stability in the three-dimensional structure were calculated using the integrated approach DUET (17), when a wild-type PDB structure is available. The effects of the mutations on the affinity of a protein–protein interface were predicted by mCSM-PPI (18) when a single-point mutation was within 5 Å of a biologically relevant interface, as defined by the author-assigned assemblies.

**Figure 1.**
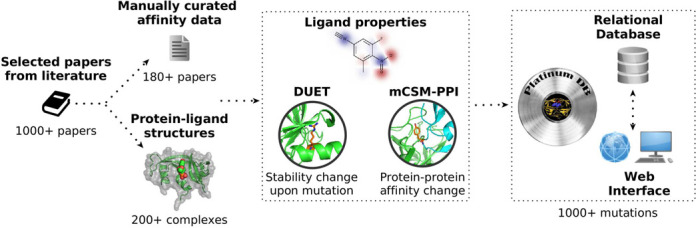
Architecture of data integration and curation of Platinum.

Ligand properties such as molecular weight, logP, number of hydrogen acceptors and donors were calculated using RDKit and complementary ligand information such as Canonical Smiles and ligand type were obtained from the PDBeChem (22). All entries were manually double-checked, with additional filters used to confirm the correctness of all structural and affinity information. Figure 1 shows the workflow for data collection and curation used to build Platinum.

### Database architecture and web interface

The collected data were consolidated as a MySQL relational database (version 5.5.35). The information stored in Platinum can be easily queried and downloaded via a user-friendly web interface. Its front-end was implemented using the Bootstrap framework version 2.0, while the back-end was built in Python via the Flask framework (version 0.10.1), running on a Linux server.

## RESULTS: DATABASE FEATURES

### Web interface

**Figure 2.**
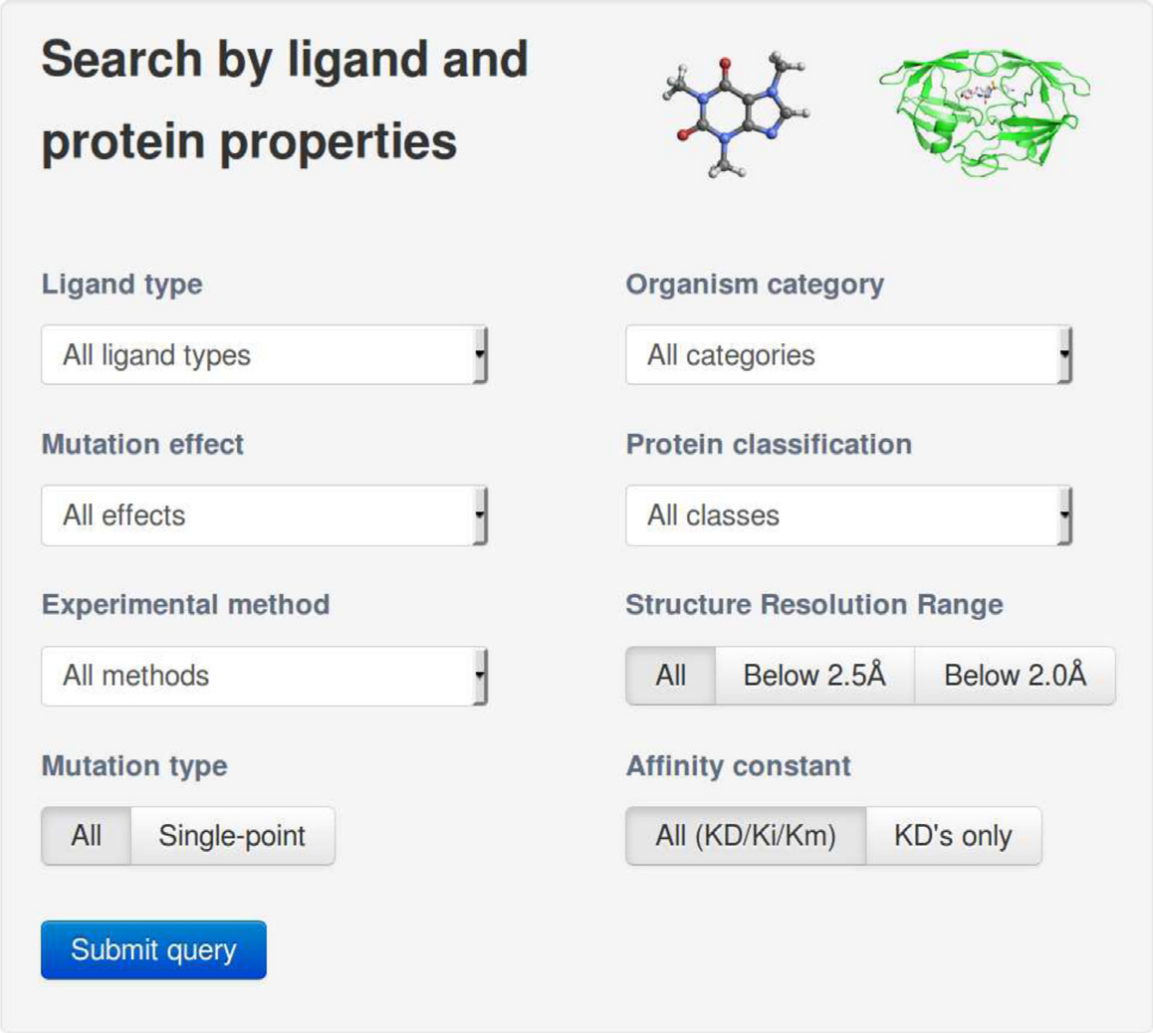
Platinums web interface search page. It allows users to query the database by combining different searching criteria such as ligand type, organism from which the protein originates and functional classification as well as mutation properties.

The web interface to the database displays the home page of Platinum. From here users can access the entire database through a ‘Browse’ function or query the database through a ‘Search’ function (Figure 2). For each data point, displayed by default (Supplementary Figure S1 in Supplementary Material) are the protein name, mutation details, whether the mutation is within 5 Å of the ligand-binding site, the ligand ID (as appears in the PDB), the type of affinity measurement, the affinity for the ligand of the reference and mutant proteins (nM), the PDB codes of the reference and/or mutant protein in complex with the ligand and the PubMed ID of the paper where the affinity data were published.

Additional information regarding the mutation, protein, ligand and affinity measurements can be toggled on and off depending on requirements of the user. Detailed information of thelocation of the mutation, including secondary structure, hydrogen bonding and solvent accessibility of the reference residue, can be displayed by toggling on residue properties. The extra experimental information includes the method, pH and temperature at which the affinities were measured, as well as the predicted changes in the affinity. The ‘protein toggle’ reveals information about the structure, including the organism, resolution and R-factors. The ‘ligand toggle’ will show several properties calculated using RDKit or obtained from PDBeChem. To facilitate downstream analysis, hyperlinks to the RCSB PDB entries and PubMed abstracts have been included.

### Querying the database and downloading the results

The database can be queried using the web interface (Figure 2). This provides the ability to search the database by ligand and protein type, the kingdom of the organism from which the protein originates, the effect of the mutation on the affinity for the ligand, how the affinity was measured and whether only high-resolution structures, *K*_D_'s or single-point mutations should be displayed.

The web interface also provides several methods for exporting data. Firstly, the entire platinum database can be downloaded as a comma-delimited file. All the processed and filtered PDB files linked in Platinum can also be downloaded as a separate file. Secondly, the results retrieved by a query can be exported separately as a comma-delimited file.

### Data statistics

**Figure 3.**
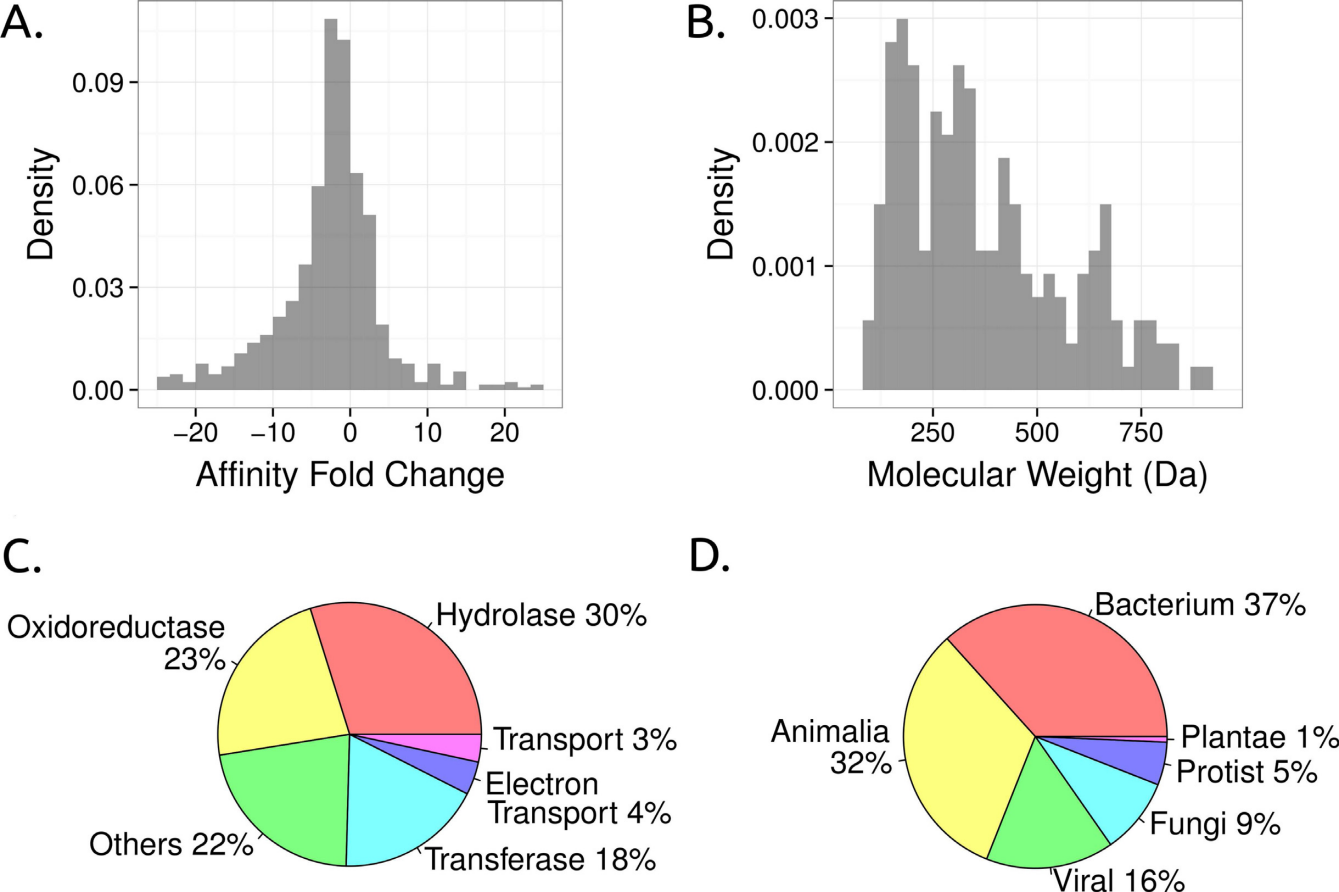
Platinum entries statistics. In (**A**), the histogram of the density distribution of the effect of mutations on protein–ligand affinity within Platinum is shown as the fold change (ratio between affinities of reference and mutant). In (**B**), the histogram of the density distribution of molecular weights of unique ligands in Platinum is shown. The proteins in Platinum are classified by their function, with the proportion of proteins in the most common classes shown in (**C**). The proteins are also classified phylogenetically in groups and the proportion of data points per class is shown in (**D**).

Currently Platinum contains more than 1000 unique data points, with 72% of mutations leading to either a significant increase (16%) or decrease (56%) in protein–ligand affinity of over 2-fold (Figure 3A). Most mutations in Platinum (75%) involve residues directly interacting with the ligand in the three-dimensional structure. Approximately 80% of the data points are from single-point mutations, which are more amenable to computational predictions. The information stored in the database represents a diverse range of proteins, ligands and interactions, as summarized in Table 1. The ligands in the database are quite varied, with molecular weights ranging from 90 to 900 Da (Figure 3B) and including fragments, inhibitors and therapeutics as well as natural co-factors and substrates (other properties of ligands in Platinum are also depicted in Supplementary Figure S2 of Supplementary Material). The proteins present in the database represent a wide range of biological activities (Figure 3C) highlighting the broad range of effects encompassed within Platinum. While the proteins are from a diverse selection of organisms, the majority (60%) are from organisms in the Bacterial Domain and Animalia Kingdom (Figure 3D), with approximately 20% of the data from *Homo sapiens* proteins. This reflects the research emphasis within these areas.

**Table 1.** Overview of data represented in Platinum

Property	Frequency
#Mutations	1008
#Single-point mutations	797
#Papers (by PMID)	182
#Unique Uniprots	142
#Unique ligands	207
#Unique protein-ligand complexes	250
#Total unique PDB IDs	451
#Affinities given in *K*_D_	560

## DISCUSSION

The threat of resistance to current therapies is becoming of increasing concern and is widely publicized, especially in regard to the use of antibiotics (23) and emerging pandemics including those caused by the influenza virus (4), in infectious diseases such as tuberculosis (24), in cancer (3) and many other life-threatening illnesses. A repository of mutations and the protein structures within which they occur should be a useful tool to aid the understanding of ligand binding, the development of resistance or the target of a drug or chemical tool. However, no attempt has been made previously to catalog these results, and thus this useful information is currently difficult to access.

Previous resources such as PDBbind (25), AffinDB (26) and BindingMOAD (27) provide only the affinity for crystal complexes, while Platinum also maps the effects of mutations on binding affinity. Platinum is also significantly different from BindingDB (28)—only 25% of affinities in Platinum are also present in BindingDB.

Platinum is the first comprehensive database of its kind that provides experimental information related to changes in protein–ligand affinities upon mutation and their three-dimensional structures. Besides providing a collection of affinity and structural data, various analyses and links to web-based databases have been integrated into Platinum to facilitate the detailed examination of these mutations. Platinum will also be useful for developing novel *in silico* predictive approaches.

We have observed that predicted changes in protein stability and protein–protein affinity upon mutation show a poor correlation with experimentally measured changes in ligand affinity; therefore a new general method to encompass these effects is required. Some properties appear to correlate better with affinity change, including the distance of the mutated residue to the binding site as well as changes in residue properties. We are currently using structural signatures together with these observations to develop a universal predictive method. The accurate prediction of the changes in ligand affinity upon mutation may not only enhance our understanding of ligand binding and aid in the drug development process but also allow directed administration of therapeutics most likely to be efficacious.

## SUPPLEMENTARY DATA

Supplementary Data are available at NAR Online.
